# Families and Choice: Disassembling Same-Sex Families with Children

**DOI:** 10.1007/s10508-026-03421-5

**Published:** 2026-06-09

**Authors:** Daphna Birenbaum-Carmeli, Daniel Sperling

**Affiliations:** The Cheryl Spencer Department of Nursing, University of Haifa, 199 Aba Khoushy Ave., Mount Carmel, 3498838 Haifa, Israel

**Keywords:** Same-sex families, Genetics, Legal parentage, Couples, Parenting, Sexual Orientation

## Abstract

With the proliferation of same-sex families, same-sex separation has expanded, creating diverse parenthood-related issues. Yet, little is known about how these family dissolutions are perceived and enacted by parents and children. This article reports on a descriptive qualitative study under the constructive phenomenological approach conducted in Israel that explores the disassembling of families of choice and its impact on involved parties. The study examined choice as construed in 20 cases, narrated in 18 interviews with lesbian and gay separated parents, supplemented by two case outlines from a psychotherapist treating separated gay couples with children. Three major themes emerged: parents choose parents; children choose parents; and parents choose children. The study reveals how genetic relatedness was taken for granted as the most solid basis for parenthood, granting parents greatest privileges and widest choice range. However, it also shows that the prominence of genetic relatedness surfaced when relationships deteriorated. Legal parenthood provided another solid foundation, going nearly unchallenged through separation. Social parenting proved the most uncertain, where parents lacked genetic or legal ties and contributed “solely” to childrearing. Despite these complexities, separated couples aspired to create families that would be largely considered normative by local middle-class terms, while maintaining tight relations with their families of origin.

## Introduction

Since Weston’s (1991) elaboration of the concept “families of choice” in 1991, the notion has been strongly—if not exclusively—associated with LGBTQ+ families (Donovan et al., 2001; Jones-Wild, 2012). Being often contrasted with genetically related heteronormative families, the term “families of choice” usually denotes enduring and dependable social ties considered as one’s relatives, effectively substituting biological kin. The concept has been part of the critique of the presumably universal “natural” genetic heteronormativity that underlies traditional kinship definitions (Strathern, 1992). The power of families of choice has been highlighted in a variety of contexts, from creating such families through assisted reproductive technologies (Heinemann, 2018) to caring for people living with HIV/AIDS (Grant et al., 2013), in older age (Cottrell, 2020; Wardecker & Matsick, 2020) and at end of life (de Vries et al., 2019). Discussions of these families have addressed their function as long-term caregivers (Washington et al., 2015) as well as in contexts which curtail one’s choice by the law (Drapalska-Grochowicz, 2023; Mizielińska & Stasińska, 2023).

Same-sex couples acknowledge the importance of chosen families in their lives (Hull & Ortyl, 2019). These couples resemble their heterosexual counterparts in various respects, being affected by similar structural factors (Kurdek, 2005), and parenthood goals (Lewin, 2019). At the same time, they also have some particular characteristics. The literature suggests that same-sex female partners spend more time together than male or different-sex couples. It also reports more diverse and equal gendered divisions of labor (Beban & Roberts, 2024; Van der Vleuten et al., 2021), Despite facing unique challenges (Hatch et al., 2021) and receiving less support from their families of origin (Church, 2020), these couples sustain similar levels of relationship quality. More generally, it has been argued that “gay kinship” does not comprise an alternative kinship paradigm, though it is often non-biological (Smietana et al., 2018).

Notwithstanding some structural similarities, same-sex families differ from other “new” families in three distinctive features: diversity, genetic partiality, and weaker legal regulation. In contrast to the monolithic heteronormative family model of a woman, a man and their genetically related offspring, families of choice, especially LGBTQ families, are diverse, lacking an encompassing hegemonic model, i.e., possessing strong influence or authority over others (Adler & Lenz, 2023; Mezey, 2015; Newman, 2019). Families headed by lesbian partners may be founded by a variety of methods, including privately arranged sperm donation from a known man or a commercial sperm bank. The egg used may be that of the conceiving woman or her partner’s. Co-parenthood with a man is another option, with varying degrees of involvement on the part of each genetic parent and their same-sex partners, if exist. Co-parenthood families may thus include two, three, or four parents, depending on the circumstances. Gay men can also opt for various pathways, mainly, co-parenthood with a woman or contracting a paid or altruistic gestational carrier, who would use her own egg or that of a donor, familiar or anonymous, to be fertilized by one man’s sperm or that of both men. The resulting embryos may therefore be identified as genetically related to one partner, or else unknown unless tested. Additionally, same-sex partners can opt for adoption, though at present, numerous countries have made adoption by same-sex couples or individuals, highly challenging (Goldberg et al., 2019; Mallon, 2011). This variability is at the core of chosen families formed by gay and lesbian persons.

A second significant difference is that of genetic partiality. In the heteronormative family model, the heterosexual intercourse is construed as a key to “interweaving love and blood symbolism, converting love ties into bio-relatedness through the alchemy of intercourse” (Ziv, 2020, p. 136). Though in many heterosexual families not all children are genetically related to both parents, the possibility of full genetic relatedness underlies the hegemonic model, as the ideal against which families are valued and legitimized. In contradistinction, in same-sex families—unless one partner is a transgender—children are genetically related to one parent at most. Fresh arguments, based on recent understandings in the philosophy of biology, support new forms of kinship, e.g., based on joint determination by multiple causes, extended inheritance, and development as construction (Maung, 2021). Still, great import continues to be bestowed on “blood ties” as basis for kin relations in Euro-American culture (e.g., Nelkin & Lindee, 2004; Schneider, 1984; Van Wichelen & De Leeuw, 2022).

The third distinctive characteristic is the looser and at times absent legal regulation of families of choice (Naaman, 2022; Park et al., 2016) leading to both legal vulnerability and barriers (Kazyak et al., 2018; Siegel et al., 2021). In many countries, same-sex couples may be registered as married and may formally divorce. Still, in numerous countries, such options are unavailable. Notably, even where possible, gay and lesbian partners often found families without a formalizing union (Badgett et al., 2025). The resulting families are therefore, less tightly regulated than heteronormative families. This gap is especially evident in countries where the majority of heteronormative couples do marry formally. The legal looseness embodies and perpetuates a marginal status, while also granting greater freedom when forming and disassembling families, enabling lesbian and gay couples to forge a wider, more personally appropriate post-separation arrangements.

### Making and Breaking Queer Families

Families with children founded by same-sex partners are not a self-evident phenomenon (Smietana et al., 2018). The proliferation of such families has been largely driven by technological advancements, consumerism, shifts in societal norms, and the separation between sex and reproduction (Mamo, 2007; Thompson, 2005). As such, parenting within these families challenges dominant discourses and culturally accepted understandings of parenthood and family (Reed, 2020), defying the taken for granted universality of genetics centered heteronormative, cisgender reproductions (Lewis, 2021). This discourse mostly reflects Anglo-American geopolitical privilege but also criticizes the rights that are associated with queer kinship (Mizielińska, 2022). Still, same-sex family founding appears to be increasingly normalized worldwide (Stacey, 2018).

Much literature has probed the making of non-traditional families (e.g., Howell, 2009; Lewin, 2023; Stacey, 2011; Thompson, 2005(. Alongside greater variability in family formation, the quest for biogenetic relatedness seems prominent also among same-sex persons, especially those employing assisted reproduction technologies (Bergman et al., 2010; Nahman, 2013; Ziv & Freund-Eschar, 2015). This might manifest in selecting a gamete donor that resembles the non-genetic parent or selecting the same gamete donor for the couple’s future pregnancies (Ziv, 2020).^1^ Recent estimates, which may be deficient, report that 18% of US same-sex couple households include children under 18 (Wilson & Bouton, 2024). Moreover, same-sex partners who consider themselves spouses are more than twice as likely to be raising biological, step, or adopted children vs. unmarried same-sex couples (31% vs 14%, respectively) (Gates, 2013). According to the 2022 American Community Survey, 22.6% of lesbian couples and 5.7% of gay couples raise children (US Census Bureau, 2024). Between 2008 and 2022, the percentage of same-sex households with children under 18 has grown from 20.5% to 27.8%. Growing prevalence of same-sex families with children was reported also form Australia (Gahan, 2017), a phenomenon dubbed the “gayby boom” (Dempsey, 2014). Adopting one’s partner’s children has also become increasingly common (Bowen, 2008; Margalit, 2012; Wardle, 2010).

With the proliferation of same-sex families, same-sex separation has also expanded. The scarce existing evidence suggests roughly ten percent incidence of family break-up among same-sex couples (Manning & Joyner, 2018), with possible greater prevalence among lesbian headed families (e.g., Andersson et al., 2004; Birenbaum-Carmeli, 2020; Manning & Joyner, 2018; Manning & Joyner, 2018; Savage, 2021). A recent survey reported a similar rate of separation among LGBTQ parents and straight women, with both groups having a higher rate than straight men (Wilson & Bouton, 2024). However, the evidence regarding relative prevalence vis-à-vis heteronormative break-up is mixed (e.g., Kolk & Andersson, 2020; Lau, 2012; Manning & Joyner, 2018). As many gay and lesbian couples do not formalize their union, it is hard to assess the scope of the phenomenon (Gahan, 2018; Gartrell et al., 2005, 2006, 2011; Goldberg & Allen, 2013; Goldberg & Romero, 2018; Turteltaub, 2002), which has been understudied to date (Yep et al., 2018).

Same-sex couples are at higher risk for dissolution the other-sex partnerships. Same-sex couples with children are more likely to get married or stay married (Farr et al., 2020), with higher dissolution risk to female couples (Scott et al., 2022). Various explanations have been suggested (Holland & Lannutti, 2023), including lower rates of legal marriages (Merteau, 2019), the relative weakness of registered partnerships vs. marriages (Chen & van Ours, 2020, Rostosky & Riggle, 2018, Farr &Goldberg, 2018), blurred expectations from one’s partner (Lerch, 2007), and wider age and sociodemographic gaps between partners (Marteau, 2019). In lesbian couples, women’s greater sensitivity to the quality of the relationship (Kalmijn & Poortman, 2006; Kurdek, 2005), much disagreement and conflict, mental health problems, infidelity, sexual dissatisfaction (Scott et al., 2022) moving in together shortly after starting the relationship (Lau, 2012; Rault, 2019), and, at times, adoption (Goldberg & Garcia, 2015) have been underscored. The fewer institutional barriers to same-sex separation (Balsam et al., 2017) may also result in higher break-up rates.

The significance of the dissimilarity in the parents’ relatedness to the children is unclear. Some same-sex parents naturalize genetic ties as automatically establishing kinship; others naturalize volition, thereby dismissing the role of third parties, namely gamete donors or gestational surrogates (Smietana et al., 2018). Officially, the difference between the genetic and non-genetic parents is often reflected in parents’ respective legal statuses: biological parent are registered upon the baby’s birth, while their partners normally must undergo a legal procedure—sometimes heavy and expensive—in order to establish their parental status legally. Where such a procedure is not conducted, non-genetic parents depend on genetic parents for their ability to maintain relations with the child, while genetic parents depend on the other parent’s will to sustain their parental commitment. In courts, non-genetic parents are often at a disadvantage. When the child was conceived via co-parenthood, the partners of the biological parents often cannot apply for parental status, as many countries outlaw more than two parents. The resulting vulnerability may underlie the readiness of many non-genetic parents to accept visibly unequal post-separation agreements (Allen, 2007; Goldberg & Allen, 2013; Turtletaub, 2002)). Non-genetic parents who had adopted their children are, however, likely to obtain more equal custody arrangements and maintain closer ties with their children (Far, [Bibr CR140][Bibr CR141]; Gartrell et al., 2011).

### The Research Field: LGBTQ+ Families in Israel

Parenthood is a crucial aspect of a person’s identity In Israel (Berkovitch, 1997, Birenbaum-Carmeli, 2004; Birenbaum-Carmeli & Carmeli, 2009; Tsfati & Ben Ari, 2018). State policies, court rulings, and religious practices all convey institutional encouragement for childbirth (Birenbaum-Carmeli & Carmeli 2009, 2016; Sperling, 2010; Waldman, 2006). Moreover, genetic relatedness is openly prioritized, as inscribed in policies and practices: Israel is the world’s only country that funds practically unlimited IVF treatment to all women younger than 45, while allocating no state support to adoption (Birenbaum-Carmeli & Carmeli, 2010; Carmeli & Birenbaum-Carmeli, 2000).

Same-sex parenthood has also gained increasing legitimacy (Golani et al., 2024; Knoll & Moreno, 2020) and became crucial for many gay and lesbian persons (Shenkman et al., 2022), resulting in exceptional rates of same-sex parenthood (Birenbaum-Carmeli & Montebruno, 2019). LGBTQ persons in Israel regard parenthood, even lone one, as empowering and strongly aspire for a biological child (Tsfati & Segal-Engelchin, 2024b). This quest for parenthood has been viewed as part of the desire for social acceptance and the local homonormativity (Duggan, 2002, 2003). Israeli gay men and their parents attributed supreme import to biological fatherhood (Tsfati & Segal-Engelchin, 2024a; Ziv & Freund-Eschar, 2015), and gay fathers ranked higher than non-parents on both “meaning in life” and subjective well-being (Shenkman & Shmotkin, 2014). Studies noted fathers’ emotional upheaval, joy and love in early parenting (Kelly et al., 2023) as well as stable and frequent contact of LGBTQ parents with their own parents (Cohn-Schwartz et al., 2023; Sperling, 2024), manifesting in intense grandparenting experiences (Tsfati & Segal-Engelchin, 2024a).

Israel demonstrates an ambiguous approach to same-sex families. While same-sex marriage is prohibited domestically, only religious marriage is permitted. Thus, marriages performed abroad can be registered locally. Lesbian women can access sperm banks, state-funded IVF, and gestational surrogacy; gay men were granted surrogacy access only in 2021. Gay individuals or couples bringing children via cross-border surrogacy must undergo strict state-controlled genetic confirmation of paternal relatedness. Same-sex couples receive full parental rights, including parental leave, child allowance, and tax exemptions. Some scholars interpret this selective gay-friendliness as "homonationalism," promoting Israel’s international image as open and liberal (Gross, 2017).Obstacles to same-sex parenthood abound. Since egg donors are scarce in Israel and donor eggs or embryos cannot be imported, gay men must seek treatment abroad. Israeli law recognizes only two parents, excluding partners of biological parents in co-parenting families. Known sperm donations are prohibited, unlike in the UK and some US states. Female partners cannot have one partner’s ovum implanted in the other’s womb, which could create two biological mothers. Domestic adoption of healthy newborns is extremely rare, and adoption by same-sex couples is virtually non-existent.

Bureaucratic hurdles compound these barriers. Non-genetic lesbian mothers cannot be registered as mothers at the hospital, unlike heterosexual parents using donor sperm. Gay men returning with babies from cross-border surrogacy must undergo state-supervised paternity testing at designated laboratories for admission and registration. Non-genetic fathers must pursue legal procedures for parental orders; adoption takes longer. These obstacles are particularly striking given the state’s disregard for genetic ties in some heteronormative families (Birenbaum-Carmeli & Rudrrapa, 2022; Naaman, 2022).

Legal parenthood is overall firm in Israel. Local courts have reaffirmed the parental rights of non-genetic parents even when partners separated before the birth, clarifying that “legal parenthood, just like biological parenthood, cannot be severed” (Peleg, 2022; Peleg & Maanit, 2022). The Supreme Court also established parental order retroactively, to the moment the parent–child relations with the gestational carrier has been severed. In one case, the court allowed estranged gay partners to adopt each other’s child after the couple had separated. In another case, the court declined a non-genetic father’s request to revoke his existing parental order and terminate his obligation to the child. The decision enhanced the legitimacy and firmness of legal same-sex parenthood, though the very consideration of the request was destabilizing. Indeed, in another case, a local court canceled adoption by a lesbian mother. Though both mothers sought this cancellation, critics argued that the ruling downgraded same-sex adoptive parenthood.

### The Research Question

While same-sex partnership and reproduction proliferate in Israel, little is known about the dissolution of such bonds. The present study seeks to explore the disassembling of families of choice, and its impact on the parties involved. The study therefore addressed the following question: how do same-sex partners enact their parenthood—genetic and non-genetic alike—throughout family separation and what can we learn from these enactments on prevailing perceptions of kinship?

This article reports on a descriptive qualitative study under the constructive phenomenological approach aiming to put together an uncovered and nuanced depiction of disassembling same-sex families with children (Schreuer & Dorot, 2017). Taking a constructionist theoretical perspective, i.e., assuming that people’s perceptions of reality as well as their social relations are socially negotiated (Gabb, 2005), the study traces the issue of choice as it is construed in participants’ accounts and the actual acts they described. These descriptions and acts, presented through semi-structured interviews, are assumed to express the contextualized significance that the participants attribute to choice in the process of family break-up. The study followed the Consolidated Criteria for Reporting Qualitative Research (COREQ) guidelines to ensure methodological rigor.

## Method

### Participants

The research population are gay and lesbian persons living in Israel. The inclusion criteria were: (1) gay and lesbian persons living in Israel; (2) who had one or more children together; and (3) parted after raising their children together. Participants’ recruitment was done via snowball communication, mainly through approaching colleagues, friends and relatives, asking about potential participants. Eight participants, all women, were recruited by previous participants’ linking. In every family, only one partner was interviewed. In the first five interviews, participants were asked if their former partner could be approached. After five consecutive refusals, this response appeared to be a structural feature, and the question was not posed anymore. Toward the end of the interview phase, the first author was referred to a woman, whom she identified as the former partner of a participant. This referral was not pursued out of respect for the previous participants’ preference.

Recruiting participants for the study proved challenging. The relative rarity of the scrutinized phenomenon might have been one reason. Many of the participants, especially the gay men, did not know other gay couples with children that had parted. Two interviewees had an indirect knowledge but were not comfortable to approach people they knew only superficially. Another probable hindrance might be the sensitivity of family separation in lesbian and gay communities. Indeed, five gay men who had separated declined the invitation to take part in the study. The refusal might have captured a reluctance to revive a painful life chapter. On a wider scale, it might have stemmed from unwillingness to stain the public image of same-sex families that has been accepted after long struggles. Indeed, elsewhere, similar reluctance to participate in studies was noted (Gahan, 2018; Turtletaub, 2002). The relatively closely knit gay community in Israel might have exacerbated concerns regarding anonymity.

Last, in order to expand the study’s sample, early in the data collection phase, author 1 approached a psychotherapist who had attended to several gay couples who had separated after having children together. The therapist offered accounts of two cases of separation in gay families with children. The conversation with the psychotherapist took place in the clinic and lasted over three hours. Having removed all identifying information, he described the couples’ roads to separation and custody arrangements. These descriptions provided supplementary data for the analysis. Interview data gathering ended upon reaching thematic saturation, signifying that additional interviews were not yielding novel or significant findings so that the issues raised in the data gained comprehensive understanding (Henneck & Kaiser, 2022; Lowe et al., 2018). Sample size was determined also by other considerations (Dworkin, 2024), such as the authors’ experience, the amount of useful information needed from each participant, and the nature and complexity of the topic resulting in practical recruitment issues, specifically when additional gay couples meeting the inclusion criteria could not be recruited, and when lesbian participants started referring to each other’s former partners, which we needed to avoid. Altogether, the present paper is based on 20 cases: 18 interviews with lesbian and gay parents who have separated from the partner with whom they have had children, supplemented by two case outlines recounted by the psychotherapist.

### Measures and Procedure

The interviews were semi-structured, person-centered, and experiential. They took place between October 2017 and June 2020. Fifteen interviews were conducted in person at a place of the interviewee’s choice and the last three were performed online by zoom, during the Covid-19 pandemic. The first author conducted 16 of the interviews (15 in person and one online). The last two interviews—both conducted via zoom—were carried out by a graduate student, instructed and supervised by the first author. Interviews lasted 1–1.5 h, with three exceptions lasting 2–4.5 h. Every interviewee was given a detailed explanation about the study and signed an informed consent form. Interviews were recorded and transcribed. The two additional cases were recounted by a psychotherapist who worked a lot with LGBTQ individuals. This therapist was willing to share the stories of two of his gay patients who separated from their partners after having had children together. Given the difficulty of recruiting participants, especially gay participants, this opportunity was much appreciated. These cases are, evidently, less detailed than the interview-based ones.

### The Interview Guide

The interview guide consisted of open-ended questions. These questions addressed one’s earlier biography and extended family history; sexual identity and outing; significant partner relations; history of relations with one’s former partner; the route to parenthood; early parenthood years; parenthood-related strengths and challenges; social interaction with relatives, friends, colleagues; the road to separation; forming the separation and custody agreements; the actual separation process; and post-separation life. Additional specific questions were added throughout the interview, in order to elicit a more nuanced picture of every speaker’s life. Sociodemographic details (age, birthplace, ethnicity, religion, education, employment, current residence) were also recorded. Participants were invited to share concrete real-life situations, thereby grounding and potentially also countering self-declared generalizations. The substantive questions were all presented in an open style, mostly asking “can you tell me how you met?” or “how did they respond?” the patient active listening apparently called for detailed descriptions and extended sharing, as was evident by the length of the interviews. The term “choice” has never been used in any question. The interview guide is attached in the Appendix.

### Data Analysis

Once all interviews have been transcribed, each interview was read closely and coded for significant motifs, which were then gathered into cross-case themes (Denzin & Lincoln, 2011). Coding was carried out by author 1 within the broader approach of an abductive analysis, i.e., constant move through the interview materials and relevant theoretical frameworks, to produce empirically grounded understandings. Under such a process, the researcher tentatively views the data through different theoretical "cases" to see which provides the best fit by questioning the taken-for-granted categories of the research field and looking for signs and variations in the phenomenon across different contexts (Tavory & Timmermans, 2014). The intermeshed, repeated, close reading and coding of the interview transcripts, alongside theoretical material, enabled the formation of broader themes. Following open and axial coding and tentative theoretical fit performed by the first author, both authors engaged in iterative discussions concerning the main themes and their subdivision into subcategories to increase trustworthiness (Stahl & King, 2020). This collaborative analytical process involved systematic comparison of coding schemes, critical examination of theme boundaries, and deliberation on the relationships between categories. Through this reflexive dialogue, the authors scrutinized emergent patterns, challenged initial interpretations, and explored alternative explanations for the data. This iterative process continued until both authors achieved consensus on the findings, ensuring that the final thematic framework accurately represented the data while maintaining analytical rigor and interpretive credibility. During article coding, relevant segments were translated into English.

### Ethical Considerations

The study was approved by the researchers’ ethics Committee at their affiliated academic institution (Application 462/18, 2018). Given the relative rarity of same-sex break-ups and the dense social network of LGBTQ parents, especially among lesbian mothers, potentially identifying details, such as participants’ gender, age, the number of the children, profession, place of residence, and religiosity, were modified. All names were pseudonymized. This adherence to guaranteeing participants’ anonymity also underlies the decision not to include a table with participants’ personal details, which might give away individuals’ identity.

## Results

The study sample included 18 persons—six gay men and 12 lesbian women—who had had children with their same-sex partners and then separated. Participants varied greatly. Four participants had one child, while others had two or three. In most families, each partner had genetically related children, but in six, only one partner had. In seven families, the children were genetically related to each other via the gamete donors; in the others, they were not. One family was founded by adoption so no members were genetically related. In the lesbian families, women conceived by donor sperm or else by co-parenthood with a man. All women conceived in Israel. In the gay families, children were either adopted abroad or else born via cross border surrogacy with the eggs being retrieved from North American or European women and transferred to gestational surrogates contracted in the USA or South East Asia. Founding gay families was, therefore, a much costlier and logistically heavier procedure.

The partners’ ages ranged from 29 to 58 at the time of the interview. The children were one to 19 years old. The separations took place two to 12 years prior to the interview. Two former partners self-described or were depicted by the psychotherapist as religiously traditional. The rest self-identified as secular. All the study’s participants, but one mother and her child, lived in urban areas in Israel. All interviewees and their former partners were employed or self-employed, and nearly all had graduate or post-graduate degrees. The others were working either in business or in the arts world. All the participants could be described as middle class, located at varying points along the category’s range. As domestic surrogacy was allowed in Israel at the time only to heterosexual couples, the participating gay couples invested in the procedure 150,000–300,000 USD, all privately funded.

The study therefore sought to answer the following question: how do same-sex partners enact their parenthood—genetic and non-genetic alike—throughout family separation and what can we learn from these enactments on prevailing perceptions of kinship?

Overall, our findings reveal that choices were central in the disassembling of one’s nuclear family. Notably, partners often differed greatly in the power to make choices and at times, the less advantaged side, most frequently the non-genetic parent, was compelled to accept uncomfortable agreements. Generally speaking, one’s freedom to choose paralleled their partner’s risk of being chosen out, of being “De-Selected." This state of affairs, which may prevail also in heterosexual separations, appeared to be especially intense in those families, where the non-genetic parent has not legally established their parental status. Below we describe the study’s three main themes: parents choose parents; children choose parents; and parents choose children.

### Parents Choose Parents

The very decision to separate is evidently an act of choice. This selective process has some particularities in same-sex families. In some couples, the intended genetic father grappled with prolong conception attempts without the support of his partner, the intended non-genetic father. Michael, Omer’s genetic father, described how he had tried to engage his partner, Tom, in the three year-long surrogacy process. Tom, on his part, refused to sign any conception related forms, before or after pregnancy. However, once Omer was born, Tom “fell in love,” and immersed in childcare, hardly letting Michael attend to the baby. At first, Michael was elated about the emerging bond, but gradually he felt that he was being pushed out of his parental position. Refusing to admit the state into his private life, Tom ruled out issuing a parental order. The men’s relationship deteriorated. Both acted as Omer’s fathers, but Michael fulfilled a greater and more consistent role and eventually decided he wanted to part. At that point, Tom approached the court to seek parental order, but was rejected as he had not previously signed any parenthood-related document. Michael was extremely frustrated by Tom’s conduct. Still, after some four years of court procedures and interpersonal fluctuations, he acknowledged that.

…eventually, I’ll have to swap sides and support him in his claim against the state, so he can be registered as a father. He is Omer’s father. It makes no sense.

In the Amotz family, each father had adopted one child abroad, in the early 2000s. Due to legal constraints in the children’s country of origin, each man adopted as a single man, leaving his partner without legal standing vis-à-vis the child. The men decided to spare the children the public attention that might have followed a request for cross adoptions that was exceptional at the time. Six years after the couple had separated, after several lesbian women had approached the court and had been granted the right to cross adopt their children, the fathers chose to pursue mutual adoption, becoming fathers of each other’s children. Ben described the fathers’ joint action and its recognition in court:

We worried that the court would not ratify post-separation adoption, but we were surprised, favorably: the Judges acknowledged that we had been together since the children were adopted and considered cross adoption as the children’s best interest. In fact, they said that adoption was especially important because we had separated.

Tightening the ex-partner’s parental bond was a rare choice, though. In several families, where co-parents were still negotiating legal parenthood statuses, the genetic parents chose to distance their ex-partners from legal parenthood. Merav and Ayala had both tried to conceive. Eventually, Merav gave birth to the couple’s two children, Joel and Neta. She also functioned as their primary carer, despite her senior professional career. Ayala intensively focused on her work and separate social life. When the children were 5 and 2 years old, the couple decided to separate. The children went together between the mothers’ homes, spending most of their time with Merav. Merav reflected on her parental privilege as the children’s genetic mother and on her emotions regarding Ayala’s motherhood:

I’ve never really accepted her motherhood. It bothers me that the kids call her mom… I don’t think it’s fair, but in my fantasy, I would love to toss her right out of my life… [but] it’s my responsibility to work on myself and accept [Ayala’s motherhood]. … it was always on the table, that I wouldn’t [disconnect her from the children], even if I said so in moments of rage. In the break-up mediation, I said that adoption must be part of the separation package... but today, I would not pursue adoption. I wouldn’t depend on her consent, for example to psychological treatment, to which she objected and did not pay anything.

Explicating her changing choices, Merav articulated her outlook on the social standing of lesbian parenthood:

There is something inherently unfair in this relationship... I have the illusion that the power is in my hands, because I gave birth and she has no genetic connection. And that’s not right…. because she’s their mother. She was there, she raised them. I don’t like it, but it’s irrelevant…But if you don’t settle the registration right after birth, it gets contaminated with a lot of emotions and fantasies. Why should the registration of lesbians’ babies differ from egg or sperm donation? I think it’s because the lesbian family is not as socially legitimate.

Merav’s introspection thus reveals her ambivalence regarding the privilege of the genetic mother. While viewing it as unfair, she still takes advantage of her privilege, by choosing to bloc Ayala’s route to adoption. In contrast to Michael, who had first materialized his privilege to stop Tom from obtaining a parental order but then decided to support his application, and unlike the two adoptive fathers, Merav chose, following her post-separation experience, to deny formal motherhood from her former partner.

In five additional families in the present sample—three lesbian couples and two gay couples—the genetic parent chose to discontinue the adoption or parental order application that the couple had previously planned to pursue. In all these families, the non-genetic parents kept pushing and requesting to advance the legal parenthood procedure but were met by postponements and eventual choice to deny them parental status. Netta, Nir’s genetic mother, initiated the separation when Nir was three years old and Yifat, Lenore’s genetic daughter, who share sperm donor with Nir, was three months old. Netta moved in with Zohar, her new partner, shortly after the break-up and has stayed with her since. Though Lenore and Netta had planned to pursue cross adoptions, once they had each given birth, after Yifat’s birth, Netta refused to embark on the procedure and initiated the separation soon afterward. Consequently, each mother had no formal status vis-à-vis her non-genetic child. At first, Netta and Zohar lived close to Lenore and Yifat. Five years after the separation, they moved to a further town, making visits harder and rarer. Three years later, shortly before the interview, Netta and Zohar relocated to Australia. Anguished Lenore acknowledged the opportunity and supported it, while recognizing that she had practically no influence on the choice. She described her increasing marginalization in Nir’s upbringing:

Netta came with a well-arranged set of ideas, revolving around the concept that I am not Nir’s mother anymore, that … I only pick him up from the kindergarten twice a week and at 8pm I return him to her.... obviously, my world has collapsed. I felt as if they had come to inform me that Nir had died. That was my feeling…Netta keeps close contact with Yifat. She talks with her on WhatsApp all the time. For instance, recently, Yifat broke her leg and Netta kept calling. I talk with Nir. When his birthday arrived, I asked Netta to buy balloons and cakes for him on my behalf. She also printed the greetings that I’ve sent to him and gave him the gifts.

At that stage, Lenore and Netta were cooperating. Each mother kept in touch with the other’s genetic child, though the communication was solely online. Formalizing their cross parental status has become irrelevant. Netta has effectively chose Zohar to be Nir’s mother.

In another family, where each father had one genetically related daughter via surrogacy, the men separated when the girls were a year old. The father who had initiated the break-up, decided to discontinue their cross-adoption applications. Each man resumed his own surname and removed his ex-partner’s surname from his genetic daughter’s surname. Three years later, the initiating father chose to invite his new partner to adopt his daughter, whom he had been actually raising for a couple of years.

A different choice of parents took place in two other families, formed via co-parenthood. Each mother had a female partner, and every woman had a genetic child. In both families, in the babies’ early years, the mothers were the main carers and their homes were the children’s main homes. The children spent two days a week and some weekends with their fathers. In one family, the mothers fought fiercely after the separation, until one mother stopped her genetic daughter’s visits with her former partner. At the same time, she drew much closer to her daughter’s genetic father, practically replacing her former partner with him, as a second parent. The mother recounted:

Disconnecting from Debbie, suddenly connected Jack very strongly to Anna. Once Debbie got out of the equation, Jack could come in. At first, Anna protested: “I don’t care, I want her! Pity you didn’t bring me by sperm donation. I don’t need this father!” … [But now], there is one father and one mother… Six months ago, I turned 50 and I said to myself: this is the best birthday I’ve had. I’m free. I don’t worry about Anna financially because she also has a father. Jack pays me now 3,000 shekels (≈ 1,000 USD) a month and he will leave everything to Anna. Jack works quite nearby, in the south. He has a room in my home. So twice a week he stays over with us. And once in two or three weeks, Anna goes to him for the weekend. Suddenly he can be in touch with us as much as we all want. We get along so well. We once went abroad, the three of us… I consult with him. He repairs and arranges things around my house and he is generous. We make no calculations.

Thus, from living with a female partner and raising together their two children in a two mothers’ family, with the fathers as distant secondary parents, Alona disconnected from Debbie and consequently, from Debbie’s genetic son and gradually made the choice to raise Anna with Jack, her genetic father.

### Children Choose Parents

Our study reveals that children who were older when their parents separated could participated in decisions that shaped their post-separation family lives. Perhaps because the lesbian families in our sample had older children, children’s agency was more evident in families headed by women. While still dependent on parental approval, four children from three lesbian-headed families were described by their mothers as agential and proactive in making life-shaping choices.

Gabi was seven at the time of the interview. He grew up with his genetic mother and her partner, and with his genetic father and his partner. The partners of his genetic parents have also had a genetic son. The four-parent family lived in two family homes, with the children moving between them. As each boy had a genetic mother and father, no additional parental arrangements could be established. When the boys were 4 and 7, respectively, the fathers broke up. Shortly afterward, the mothers separated. Gabi was very fond of Doron, the ex-partner of his genetic father, who was the genetic father of his own brother. As long as both couples of mothers and fathers were together, Gabi spent much time with Doron. However, after the break-ups, Gabi did not stay on his own with Doron and saw him less often. Gabi missed Doron and watched with pain how Dean, his brother, was spending much time regularly with Doron. Dikla, Gabi’s non-genetic mother, described how the 6-year old tried to influence his parents’ choices:

There’s a struggle between them [the boys] over Doron. Gabi sometimes has such questions: “Why do you go by biology?” “Why does biology determine?” We said to him that he was right, that it shouldn’t have determined, because we are all his parents, but that’s how it evolved. He envies Dean because of Doron.

Gabi’s effort was somewhat successful as it has probably influenced Doron to maintain close relations with him. His ability was, however, limited. Four of the older children whose mothers participated in the present study were, however, more capable to influence their post-separation life circumstances. Becky was one mother who shared such a description.

Becky and Dovrat had each given birth to one child and adopted each other’s genetic child. When the children were in their mid-teens, Dovrat formed a new relationship, and the couple separated. Dovrat moved in with her new partner and the children, who were shattered by the change, refused to go stay with her and went on living with Becky in the rented apartment where they had lived as a family for six years. Becky described the choices that shaped the post-separation arrangements:

After a few months of living exclusively with me, I took them to my client’s holiday home in Spain. By the time we returned, it was easier for them to go to Dovrat. Then Gili [Dovrat’s genetic daughter] started to move away from me. I have no idea why. Maybe she went through some brainwashing. Our relationship was bad then. She slept only at Dovrat’s. She would come to babysit for the neighbors above me and not knock on my door to say hello, on her way up. At the time, there were some monetary disagreements between me and Dovrat and Gili took photos of my work schedule. She asked me how much money I was making. She openly chose to side with Dovrat. That was a shock for me. I felt I needed to keep her away from me. The fifty-fifty arrangement did not work. … Ron [Becky’s genetic son] was very confused between the two homes. Dovrat was perfectly fine with both of them… At some point, I told Gili I could not take it anymore and asked her to move there [to Dovrat] permanently. Maybe it was better that every child grew up with their biological mother.

Gili’s choice to proactively take a stance against her non-genetic mother—possibly part of her adolescence years—was the only such open hostility against one’s parent reported in the present study. However, in two other female headed families, teen age children actively chose to live exclusively with their non-genetic mothers. Galia and Dotti had each given birth to a child and mutually adopted their partner’s genetic child. When they parted, Galia stayed in the family’s home, which had been purchased largely by her parents in the couple’s early days. Dotti rented an apartment nearby. The couple reached a joint custody agreement, with the children spending half their time with each mother. As she described the separation, Galia mentioned dismissively the agreement she and Dotty made at the mutual adoptions:

It [the agreement] was so silly that it said that if we split, each mother would take her own [genetic] child, which obviously wasn’t on the agenda at all… It was clear that separating the children was not an option. There was no option of tearing apart at this level, including separating any of us from the children.

After moving between the houses for several months, Dotti’s genetic son, Guy, a high school student, said that the moves were inconvenient for him and went back to his room in Galia’s home, where he had lived since he was one year old. Several months later, Riki, Galia’s genetic daughter, moved back to Galia’s home as well. Both children, already in their latter teens, met Dotti once a week or fortnight, depending on schedules. Dotti paid Galia a small sum for Riki’s upkeep, but nothing for Guy. “I didn’t ask for anything for Guy,” Galia explained, “as I was afraid she’d demand that he stayed with her.” Most weeks, schedule permitting, Dotti would pick up Riki from an afterschool class and spend the afternoon with her.

In the second family, the mothers, who had each a genetic son, took for granted that the boys would move together between the two houses. Michal, one mother, moved in with her new partner, Rona, who was just having a child via co-parenthood. Michal’s genetic son, Itay, bonded with Rona’s daughter, Orit and grew very close to Rona as well. When his genetic mother and Rona had another baby, Ronen, Michal adopted him right away. (Orit has a father so could not be adopted.) Itay subsequently withdrew from their home, effectively choosing Michal’s former partner’s place as his full-time home:

From the moment Ronen was born, Itay made his visits rarer… Now, for a very long time, he doesn’t sleep here at all…he punished me. He said so: "I want to hurt you. I’m sorry it hurts, but" ... So I end up paying Sharon [Michal’s former partner] alimony because he stays more with her. It’s a very, very hard experience, to pay her for keeping a child I don’t want to stay with her, but he wants to...

### Parents Choose Children

Finally, parents made choices during separation that shaped their future relationships with their children. In most cases, parent–child relationships were maintained, though to greatly varying degrees. These emotionally charged decisions were often complex and not clear-cut. Robby Renana was studying in the USA when he met Steve. After several years of cohabitation, the two men decided to have two children, “one each,” by surrogacy. Each father adopted his partner’s child. When the children turned four, Robby wanted to return to Israel, as previously planned. Steve was reluctant but eventually agreed and the family moved to Israel. After a few euphoric months, Steve gradually resumed his work in the American company he had worked for. He increasingly withdrew from local social life and from childcare and started drinking heavily. After some time, Steve informed Robby that he was moving to the USA permanently. Robby dwelled on Steve’s love for the children and on his fear of losing them:

See, Steve is an excellent dad. It was very difficult for him in Israel. He loves the children terribly. They have a warm relationship, still today, but they see him once every two or three months. He comes to Israel and lives here and we go there for a few weeks in the summer... He doesn’t want us to divorce... He constantly reminds me to pay his Social Security so that the state doesn’t think he has left because this may cut off his affinity to Israel and then to the children…

Eventually, Robby delved deeper into Steve’s relationships with each child:

When we fight, he often says things like: "don’t take Tom [Steve’ genetic son] away from me," and I tell him: "you’re saying a terrible thing, that I may take the kids away from you, which I’ll never do, but what about Shelly [Robby’s genetic daughter]?" He says "Shelly doesn’t need me. Tom does." He separates between them very clearly. When he comes here, he spends a lot of time with Tom and nearly ignores Shelly. [He] doesn’t try to create equality.

Steve emerged, then, as a loving father, who has nonetheless chosen to live an ocean away from his children. Though the men have adopted each other’s child, Steve invested but minimal time and money in the children’s upbringing and, according to Robby, paid visibly more attention to his genetic son, while sidelining his paternal tie with his non-genetic daughter. Notably, he had adopted his ex-partner daughter and his very fatherhood was, however, never challenged.

In the co-parenthood Arbel family mentioned above, formed by a couple of lesbian women who had a child each, with the fathers living as a gay couple, the fathers broke up and then the mothers. As no couple was married and every child had two genetic parents, the non-genetic parents had no official status vis-à-vis their non-genetic children. After the separations, the four parents set a complex weekly schedule that aimed to sustain all parent–child ties. However, the planned schedule has gradually eroded. The mothers formed a routine that secured a weekly time slot to each mother with her non-genetic child. One father was routinely visiting his non-genetic son, who lived nearby. The second father chose to move further away and maintained regular contact with his genetic son, but stopped contacting his non-genetic child, whom he met only in wider gatherings, like birthdays and holidays. In practice, he has stopped functioning as the boy’s father.

In the Halfi family, the choice was evident. Batia, Shani’s genetic mother, recalled how Nurit, her partner, right after giving birth to the couple’s second child, “desperately wanted” to pursue cross adoptions. Batia, on the other hand, was overwhelmed by the sudden change and “started making divisions and separating my Shani from her child …” The couple did not pursue adoption or parental agreement and some two years after the second birth, when Shani was ten, decided to break up.

It was clear to me from the first moment that there would be no joint custody, no one-week agreement, with all the children with me for a week and all with Nurit on the next. Rather, mine goes with me and yours go with you. We’ve never made any formal agreement. It was clear that each one takes her own…

At the time of the interview, the two women met together with their children roughly once a year. The children called their “former mothers” by their names. “There was a divide,” Batia summarized, referring to the women’s decision to enact a divide along genetic lines, thereby severing their parental ties with their non-genetic children.

In the Shlush family, each of the fathers had had a daughter by a gestational carrier, using the same donor’s eggs. Before the parental order was finalized, family problems mounted and one of the men decided to part. At that point, each man withdrew his adoption application and changed the family names so that each man and his daughter carried the genetic father’s natal surname. Joe, the interviewed father, who initiated the separation, described the ability to take the whole family apart as crucial in his choice:

We said: “We now have an opportunity to break up, let each one walk away with his daughter because they are really small. There is no real connection between the so-called sisters that we are cutting up.” That’s something we wouldn’t have done… the genetic aspect, it gave us an escape route, let’s call it… as long as the parental order was not completed–for once it’s finalized, genetics has no grip …I knew it was my last chance to dismantle this thing with each one walking away with his family cell to live his life … maybe that gave me the drive to–ok, that’s what we’ll do! …

Joe went on to describe his feelings after the decision to disconnect from Dorel and Vered:

I’d be lying if I said that I don’t miss Vered. But I did some sort of cut, in my head, in my heart, and told myself that I had the privilege of being her parent, I wouldn’t call it dad, maybe: of being a central figure in her childhood, for over a year, and now she is with her dad, in her family, where she is much loved. I’m not Vered’s father and Dorel is not Sigal’s father.

Six years later, at the time of the interview, Joe’s new partner was pursuing adoption of Sigal. After a period of complete detachment, the former partners resumed annual meetings with their daughters. They decided to tell the girls their births’ details, including their shared egg donor origin, at a suitable time in the future.

A similar logic underlay the father–child separation choice in another gay family where each partner had a one-year old genetic child. However, in this case, the parental orders were issued prior to the separation. One of the fathers approached the court and requested to cancel his parental order, claiming he was no longer the father of his non-genetic child. The court sent a social worker and a psychologist to examine the request and eventually rejected it, arguing that the child showed attachment to the father and it was in his interest to stay in touch with the father. Consequently, each man was raising his genetic child but all four met once a month and the two children stayed together with each father one weekend a month. In this case, too, each man resumed his own family name and changed his child’s name accordingly.

The last few cases demonstrate the variability in same-sex separation: whereas some parents considered the choice to split the children by their genetic origin an absurd and took for granted both partners’ parenthood and the children’s siblingship, others chose the opposite as self-evident and detached themselves from their non-genetic children. To cite Joe’s words: “it was this very option, to detach and start a new life …that made the separation option appealing.”

## Discussion

Despite the increasing prevalence of same-sex partnerships and reproduction in Israel, family dissolution in these contexts remains largely unexplored. This study addressed this gap by examining how same-sex partners—both genetic and non-genetic parents—perform and negotiate their parenthood during family separation, and what these negotiations reveal about contemporary understandings of kinship. Findings show biological connection served as the presumed cornerstone of parenthood, providing maximum authority and decision-making scope. However, genetic ties became especially significant during relationship dissolution. Legal recognition constituted another robust foundation, remaining largely unquestioned throughout separation. Social parenthood was most vulnerable when parents held neither biological nor legal status and engaged solely through caregiving. Nevertheless, separating couples aimed to form families deemed conventional by local middle-class standards while sustaining strong ties with their birth families.

The distinct and diverse characteristics of families of choice (Ammann, 2025) underlie much of the dynamics recounted in our findings. Weaker legal regulation, structural variability among families, including in the parents’ relatedness to the children, result in a less structured terrain, both allowing and enforcing greater personal choice on the separating partners. The various types of parenthood—genetic, legal, and social—each seems to nurture particular disassembling related choices.

In our study, genetic relatedness, which is also enacted in the form of routine childcare, was invariably taken for granted as the most solid basis for parenthood and granted parents the greatest privileges and widest range of choice. Participants’ descriptions suggested a rather clear pattern, in which the import of genetic relatedness was relatively marginalized before conception and in the child’s earlier years. Even the formative choice of the partner who would conceive or have his sperm used, was primarily attributed to the clinical significance of age, giving precedence to the older partner, or else, to the partner who was more keen to conceive or found a family. In all cases, however, a subsequent pregnancy by the second partner was planned. No couple in this study was planning to have one child only.

This dimming of the significance of genetic relatedness is consistent with earlier findings (e.g., Dempsey, 2014), which showed that when discussing genetic matter, i.e., whose sperm will be used, the importance of genetics was typically minimized, probably in order to constitute greater equity between the fathers (Riggs, 2018). Still, alongside constructionist (Schneider, 1984), performative (Souralová, 2019) conceptualizations of kinship that focus on actors’ situated understandings of kinship (Gabb, 2005, 2022), genetic relatedness, and the desire for a genetic offspring kept surfacing as central in couples’ reproductive choices (Biton, 2025; Maya & Ben-Ari, 2023; Nahman, 2013).

In the present study, three fathers actively aspired to belittle the social significance of genetic relatedness, claiming they had performed paternity tests only due to the state requirement. In one family, the fathers, both of whom had genetically related children, consistently condemned any preference on the part of relatives. Two other pairs of parents assumed parental responsibilities as symmetrically as they could. In another family, the parents of the non-genetic father funded most of the surrogacy procedure. Two men claimed that had it been more accessible, they would have chosen to adopt rather than pursue cross-border surrogacy. In two families, the non-genetic mother was the primary carer. All the participants hailed the importance of social kinning via routine childcare.

Nonetheless, as shown in our study, the prominence of genetic relatedness (Carsten, 2004; Franklin & McKinnon, 2001; Geerts, 2026; Howell, 2006) has surfaced when the partners’ relations deteriorated. Genetically related parents took for granted their parenthood and were also perceived by their surroundings as primary carers. This primacy of the genetic parent was indisputable. Three participants described, unprompted, how they increasingly focused their attention on their genetic children. In almost all the families in this study, genetic relatedness was the key to the separation arrangement. Gravitation toward genetic parenthood—which is sustained and privileged by social policy and public perceptions in Israel (Birenbaum-Carmeli & Rudrrapa, 2022)—was especially notable in the self-evident manner in which every partner assumed custody on his or her genetic child. Symbolically, partners who had hyphenated their surnames, resumed their maiden names and changed the children’s surnames accordingly.

Genetic relatedness in itself was, however, insufficient. All the families depicted in this study owed their very being to various third parties. Still, no parent has chosen to sustain any relationship with the gamete donor. No parent has chosen to even represent the gamete donor as a full person with continuous presence in the family’s life. Obviously, none has been construed as a parent. Some fathers chose to maintain more continuous relationships with the gestational carriers. However, in no case were these women considered as mothers by any of the parties. On the contrary, once the baby was born, the gamete donor, and at times the gestational carrier as well, was marginalized and hardly mentioned in most families’ narratives. Notably, siblings, who shared a donor genetic origin, were at times nearly or fully disconnected. This choice to minimize the significance of the “third party” in the child’s life and identity suggests that genetic relatedness in itself was insufficient as basis for parental relationships. Rather, genetic ties had to be enacted in the form of childcare to be socially charged as parenthood.

The other basis for solid parenthood as demonstrated in this study is law. Commonly applied to a person or a couple who bring an unknown, not biologically related child into their family (Howell, 2003), in the families depicted above, adoption and parental order were applied to a child who was the genetic offspring or previously adopted child of one’s partner. Partners who chose to adopt or issue parental order to their partner’s genetic child acquired state recognition in their parenthood. Moreover, legal parents have materialized their parenthood also by participation since planning the conception and throughout the child’s daily life, taking part in the effort and labor of family and kin relations, thereby forming what is known as the concept of “linked lives” with the child expressed in the spacing, density, and sequencing of parent—child relationship (Roy & Settersten, 2022). Such legal parenthood proved by and large solid and went nearly unchallenged through partners’ separation. (For similar observations see Gartrell et al., 2006.) This strength of legal parenthood might be an embodiment of the parents’ preexisting attitude or else an impact of legally formalizing one’s parental status. Either way, as shown in this study, even where the couple’s teenage daughter has drawn visibly closer to her genetic mother, and at times intentionally acted against her legal mother, none of the parties has questioned the parental status of the legal mother. Possibly, the very act of legally formalizing one’s parental status is a statement that biology is not the sole basis for parenthood, simultaneously granting childrearing an equal standing (Carsten, 2004).

Extreme and exceptional cases suggest, however, that legal parenthood may have some weaknesses. First, as long as the legal procedure has not been completed, the non-genetic parent has no protection or obligation whatsoever. Thus, our study reveals that in families that chose to break up during the legal procedure, parents were totally dependent on their ex-partner’s choices to grant access to their genetic child or to maintain their commitment to a non-genetic child. Second, legal parenthood may potentially be de-established by the court. In the present study, the father who had sought court approval to terminating his parental order and his role as a father of the genetic child of his estranged partner was refused but the very discussion of the appeal suggests that under certain circumstances, the court might have revoked legal parenthood, as it indeed has several years later (Yaron, 2020). Though the case was hailed as exceptional, its very existence is instructive. One may wonder, from a stratified reproduction perspective whether genetic parenthood is socially and morally positioned as superior to legal parenthood (Rulli, 2016). Notably, under extreme circumstances, courts may legally terminate genetic parenthood, too. Such a verdict has also been issued in Israel, in the case of a misidentified baby (Maanit, 2025).

Evidently, the most uncertain is social parenting where parents have neither genetic nor legal tie to the child and have “solely” contributed to childrearing, even for years. As shown, mothers and fathers who had raised their partners’ genetic children, including ones who were described by the genetic parents as the main caretaker, were totally unprotected during break-up. Probably referring to this sidelining of “the effort that goes into the creation of kin ties” (Carsten, 2004), a California court stated that “The true test of parenthood is the actual caretaking role and an investment in the relationship with the child” (Deprince, 2015). According to this logic, families are constituted by performative choices like hyphenating surnames, visiting clinics or selecting gamete donors together, as well as through jointly allocating family roles to extended relatives like grandparents and aunts (Finch, in Ziv, 2020).

Thus, alongside genetics and the law, caretaking is recognized, if partly, as a basis for parenthood. However, unlike adoption, which is by and large irreversible and independent of the genetic parent’s ongoing approval, social parenthood requires continued endorsement by the child’s genetic parent (Ziv, 2020). As such, it is contingent, permanently depending on the willingness of the child’s genetic parent. By the same token, a non-genetic parent who has not formalized their parental status legally, can also choose to walk away after separation, shedding off their parental obligation. Social parenthood is, therefore also terminable.

Within this complex reality, the entire terrain of kinship emerges as destabilized: not only social, but genetic and legal parenthood, could each be challenged and under certain circumstances, be severed. At the same time, none of these ties is mandatory for ongoing parental relations. Queer kinship thus appears to be both liberated and personalized but also ambiguous and vulnerable. Within this unclear landscape, ties which merged genetic or legal parenthood with intensive contact and childcare seemed to prevail across partners’ separation.

Our findings are, evidently, located within the Israeli context, with its strong emphasis on family and parenthood and where gay and lesbian families are legal, well accepted and often encouraged, like their heteronormative counterparts, to have children (Sperling, 2010). More specifically, lesbian women are entitled to funded IVF, if needed, and can use local or international sperm banks as well as state supervised surrogacy. As of January 2022, men can also contract Israeli gestational carriers. This policy is reflected in the present study: no gay family was founded by domestic surrogacy. Likewise, no lesbian couple made use of a known sperm donor or conceived with one’s partner’s ova, two practices which are illegal in Israel. For the same reason, no child had more than two formal parents. Domestic adoption was also not performed as it is extremely rare and same-sex parents are unlikely to be selected as adoptive parents. Beyond the logistic hurdles, surrogacy is generally preferred by gay couples in Israel, due to the desire to raise a healthy, un-traumatized newborn. The broader legal context within which the reported cases have unfolded, is also of relevance but merits a separate discussion.

At the broader political level, lesbian and gay couples emerge in this study as largely homonormative, affirming the heteronormative order, rather than challenging it, being chiefly centered on personal affairs and domesticity and fairly depoliticized (Boulila & Carri, 2025; Duggan, 2003). This attitude, dubbed homonationalist (Gross, 2017; Puar, 2013), probably underlay the state’s ability to recruit the local LGBTQ friendly policies in support of the nation building project, as a symbol of Israel’s liberal self-image (Gross, 2017), especially abroad. Though they are not considered a threat to the social order, local LGBTQ individuals and communities do take part in Israel’s political life (Tsfati & Ben Ari, 2018).

The couples’ embeddedness in Israeli culture is visible also in broader circles. Preference for genetic relatedness runs through Israel’s reproductive policy in many ways, such as exceptional public funding of IVF vs. lack of funding for adoption (Birenbaum-Carmeli, 2016), which cultivates a notion of Jewish Israelis as a biologically related collectivity, with “the family” as its natural building block (Birenbaum-Carmeli & Carmeli 2009, 2010; Birenbaum-Carmeli, 2016). The present article suggests that these notions reverberate among local gay and lesbian couples as well. Within this perspective, same-sex custody arrangements that underscore actively materialized genetic parenthood, may be read as an instance of homonormativity. Moreover, the emphasis on genetics and actual life sharing, may be especially suited to the state’s territorial claim for “The Land of our Forefathers”: portraying Israel’s Jewish community as both “a tribe” by genetics and a social community by lived experience. Whereas the men and women involved, most likely do not act along these political lines, their actions may still be influenced by the surrounding climate and the resulting image may still be applied as a vehicle in state politics.

To sum up, in some contrast to Weston’s observation regarding “families of choice,” and more similar to Lewin’s findings that same-sex partners seek close ties with their kin, valuing their support as “the most reliable kinds of caring that could be obtained, largely because of the “natural” bonds that blood kin have for one another” (Lewin, 2019), the couples interviewed in this study aspired to create normative families and sustained tight relations with their families of origin. At times, the closeness to one’s own relatives came at the expense of some distancing from one’s partner. Just as the children turned the couples into families (Lewin, 2019), so their disassembling tells the stories of the families’ anatomy.

### Limitations

A major limitation of the present study is the reliance on one parent for the presentation of a couple’s life and break-up, as mentioned above. Whereas this research design foregoes the quest for factual accuracy and an objective “truth,” it still voices participants’ narratives, thereby showing how they make sense of their experiences (Bruner, 1986) and order past life events coherently within broader social, economic and political contexts, as leading up to the present moment (Freeman, 2010). As such, one partner’s narrative is substantive and valuable in itself. Still, interviewing both members of the couple in the future may offer additional nuance, insights, and perspectives.

Another limitation stems from the participants’ sociodemographic makeup. Recruited via snowball referrals, the participants are almost all Jewish. (As mentioned, the only exception was not depicted as such for the sake of anonymity.) The majority were highly educated and reasonably comfortable economically. As such, the interviewees evidently do not represent the local LGBTQ community, which is much more socially diverse. Having said that, given the material and social resources that Israeli LGBTQ persons have to invest in founding a family, the demographic composition of the study’s participants is not entirely arbitrary.

The relatively small study sample, though common in qualitative studies, is an additional limitation. Despite the modest sample, the present paper hopes to provide a glance at the emerging and understudied phenomenon of separation in same-sex families with children, referring to the varied understandings of forming, being and breaking LGBTQ families.

It may also be that our study faces recall bias for individuals who broke up a long time ago. Lastly, as the study was conducted approximately five to eight years ago; however, due to the security situation and periods of instability in Israel, the findings are only being published now. In the intervening years, Israel has undergone significant political and social transformations. While homophobic rhetoric from certain political figures may suggest a potential regression in the social standing of non-traditional families, recent legal milestones indicate a move toward greater inclusion. These include the Supreme Court’s landmark decisions to grant surrogacy access to gay and single men (2021) and to allow same-sex adoption under the same conditions as heterosexual couples (2023). Furthermore, the Ministry of Health’s 2022 directives to lift restrictions on blood donations from gay and bisexual men, alongside the simplification of gender-affirming care, signal a progressive shift in the state’s approach to LGBTQ+ rights.

### Conclusions

This study sought to explore how same-sex partners perform and negotiate parenthood during family separation and the resulting implications for contemporary understandings of kinship. The research findings reveal that genetic relatedness functions as the primary foundation for parenthood, conferring the greatest privileges and choices, while legal parenthood provides another robust basis that remains largely unchallenged during separation. Conversely, social parenthood is the most precarious, especially for parents lacking genetic or legal ties whose contributions are exclusively care-based. Despite these complexities, separated couples aspire to establish normative families and maintain close relationships with their families of origin, though this closeness can sometimes come at the expense of partner relationships.

These findings highlight that just as children transform couples into families, their experiences through dissolution illuminate the fundamental structures and vulnerabilities of contemporary family formations. A significant implication for practice and knowledge is the urgent need for legal reforms that recognize diverse pathways to parenthood and protect non-biological, non-legal parents during separation. Furthermore, the study suggests that reproductive policies may inadvertently reinforce biological essentialism, which can undermine the care-based dimensions of parenthood by over-emphasizing genetic relatedness during relationship breakdowns.

To advance knowledge in this area, future research should prioritize longitudinal studies that follow families from formation through separation, as well as studies investigating children’s own perspectives and the roles of extended family networks in post-separation arrangements. While the precarious nature of social parenthood presents ongoing challenges, understanding the complex interplay of genetic, legal, social, and cultural factors becomes increasingly vital as family diversity expands. This ongoing research is essential for the development of equitable policies and support systems for all contemporary families.

## Data Availability

The data supporting the findings of this study are available upon request from the authors, subject to the confidentiality and privacy of research participants.

## Appendix: Interview Guide

### Family and Gender History

Can you please tell me about

- Your childhood
- Coming out of the closet (according to flow of conversation, ask specifically about reactions from relatives, friends, schoolmates, teachers, army acquaintances)
- Significant relationships you had
- The acquaintance and the beginning of the relationship with the partner with whom you had children (use name if already known)
- The decision to start a family and the decisions of who will be genetically related and how to conceive (joint parenting, gamete donation, etc.)
- The process of family creation (according to flow of conversation, ask specifically about conception, pregnancy, childbirth, early parenthood)
- Reactions from people around you
- Relationship challenges with your partner
- The road leading to the decision to separate
- Separation and custody arrangements (according to flow of conversation, ask specifically about financial, legal, and familial arrangements and custody of the children)
- Current relationships between the members of the split family (according to flow of conversation, ask specifically about the extended family)

### General Questions

- Do you feel any difference between your relationship with your genetically related children and with your other children?
- How do the children call you and your former partner?
- What are the children’s and parents’ surnames?
- Do you and your former partner have wills? How is the property divided among the children?

### Sociodemography

- Year of birth
- Country of birth
- Religion
- Level of religiosity
- Education
- Occupation
- Current place of residence

## Footnotes

1 A gamete is a mature male or female sex cell which is able to unite with another gamete, of the opposite sex, in sexual reproduction to form a fertilized egg.
